# Overproduction of superoxide dismutase and catalase confers cassava resistance to *Tetranychus cinnabarinus*

**DOI:** 10.1038/srep40179

**Published:** 2017-01-05

**Authors:** Fuping Lu, Xiao Liang, Hui Lu, Qian Li, Qing Chen, Peng Zhang, kaimian Li, Guanghua Liu, Wei Yan, Jiming Song, Chunfang Duan, Linhui Zhang

**Affiliations:** 1 Environment and Plant Protection Institute, Chinese Academy of Tropical Agriculture Sciences, Haikou 571101, China; 2 National Key Laboratory of Plant Molecular Genetics, CAS Center for Excellence in Molecular Plant Sciences, Institute of Plant Physiology and Ecology, Shanghai Institutes for Biological Sciences, Chinese Academy of Sciences, Shanghai 200032, China; 3 Tropical Crops Genetic Resources Institute, Chinese Academy of Tropical Agriculture Sciences, Haikou 571101, China; 4 Institute of Tropical and Sub-tropical Cash Crops, Yunnan Academy of Agricultural Sciences, Baoshan 678025, China

## Abstract

To explore the role of protective enzymes in cassava (*Manihot esculenta* Crantz) resistance to mites, transgenic cassava lines overproducing copper/zinc superoxide dismutase (*MeCu/ZnSOD*) and catalase (*MeCAT1*) were used to evaluate and molecularly confirm cassava resistance to *Tetranychus cinnabarinus.* Laboratory evaluation demonstrated that, compared with the control cultivar TMS60444 (wild type, WT), the survival, reproduction, development and activities of SOD and CAT in *T. cinnabarinus* feeding on transgenic cassava lines SC2, SC4, and SC11 significantly inhibited. Furthermore, the activities of SOD and CAT in transgenic cassava lines SC2, SC4, and SC11 damaged by *T. cinnabarinus* significantly increased. These findings were similar to the results in the mite-resistant cassava cultivars. Besides, field evaluation indicated that the transgenic cassava lines SC2, SC4, and SC11 were slightly damaged as the highly mite-resistant control C1115, while the highly mite-susceptible WT was severely damaged by *T. cinnabarinus*. Laboratory and field evaluation demonstrated that transgenic cassava lines were resistant to *T. cinnabarinus*, which directly confirmed that the increase in SOD and CAT activities was positively related to cassava resistance to *T. cinnabarinus*. These results will help in understanding the antioxidant defense responses in the cassava–mite interaction and molecular breeding of mite-resistant cassava for effective pest control.

Cassava (*Manihot esculenta* Crantz) is one of the most important root crops grown in most of Africa, Southeast Asia, and Latin America. The high starch content (20–40%), easy management, and favorable tolerance to harsh soil and climatic conditions make cassava not only a staple food for about 800 million people in the tropics^1^,^2^, but also a desirable industrial and feeding source^3^,^4^,^5^.

*Tetranychus cinnabarinus* (Boisduval), identified as a dangerous pest, has a short life cycle, rapid development, high fecundity, and wide host ranges^6^. During the serious damage period, *T. cinnabarinus* can cause leaf fall, which could bring about up to 50–70% reduction or even no harvest in cassava production. Nowadays, it has become one of the most important pests all over the world^7^. So far, the use of pesticides is the popular method to control *T. cinnabarinus*. However, *T. cinnabarinus* often outbreaks after cassava has been planted for 6–8 months when it is very difficult to spray pesticides, which results in low-efficiency use of pesticides, increase in the amount and frequency of pesticide application, and serious resistance, resurgence, and residue problems (“3 R” problem). Therefore, an effective control of *T. cinnabarinus* has become one of the most important issues to be resolved in modern cassava production today.

Much of the plant injury, including those caused via chewing and sucking by herbivores, is associated with oxidative damage at the cellular level^8^. A typical phenomenon of oxidative damage is the rapid accumulation of reactive oxygen species (ROS). ROS are highly reactive and can seriously disrupt normal metabolism through oxidative damage to lipids, protein, nucleic acids, and cells^9^, which can cause host cell death and chlorosis^10^. Several protective enzymes such as superoxide dismutase (SOD), catalase (CAT), peroxidase (POD), polyphenol oxidase (PPO), and ascorbate peroxidases (APX) are involved in ROS detoxification. The induction of these protective enzyme genes is closely related to plant resistance to herbivores. For example, the induction of the activities of SOD and CAT may contribute to bioprotection of cucumber plants against *Bemisia tabaci* infestation^11^. Similarly, enhanced activities of the protective enzymes SOD and CAT were one of the most essential elements of defense responses in pea seedling leaves to oxidative stress triggered by pea aphid (*Acyrthosiphon pisum* Harris)^12^. Maserti *et al*.^13^ found that 5 of 50 identified proteins induced by two-spotted spider mite *Tetranychus urticae* and methyl jasmonate in citrus leaves were oxidative stress–associated enzymes, including phospholipid glutathione POD, APX, Mn-SOD, and a salt stress–associated protein. Wei *et al*.^14^ found that variations in SOD, POD, phenylalanine ammonia-lyase (PAL), and PPO activities were closely correlated with alfalfa resistance to aphid and could be used as physiological indexes for testing aphid resistance of alfalfa. However, these reports lack direct and convincing evidence that the overproduction of protective enzyme genes contributes to the resistance to herbivores.

Transgenic technology is an ideal strategy for manipulating gene expression to study the specific roles of protective enzymes in scavenging ROS and other beneficial functions. Several groups have addressed the overproduction of SOD in the chloroplasts as a means to enhance tolerance to oxidative stress^15^,^16^,^17^. In addition, transgenic plants expressing CAT had increased tolerance against various abiotic stresses^18^,^19^,^20^. Gene stacking strategy in transgenic plants was also commonly employed. Transgenic potato plants expressing both SOD and APX in chloroplasts had enhanced tolerance against oxidative stress and high temperature^21^. Chinese cabbage plants expressing both SOD and CAT in chloroplasts had enhanced tolerance to sulfur dioxide and salt stress^22^. Besides, previous studies found that the overproduction of SOD and CAT not only delayed postharvest physiological deterioration of cassava storage roots^23^, but also improved tolerance against cold and drought stresses^24^.

However, the aforementioned studies mainly focused on the overproduction of protective enzymes that enhanced tolerance toward abiotic stresses. A few available reports state that the overproduction of protective enzymes contributes to plant resistance to pests. Therefore, to understand the role of protective enzymes in cassava resistance to *T. cinnabarinus*, in this study, the changes in SOD and CAT activities were analyzed in resistant and susceptible cultivars damaged by *T. cinnabarinus*; it was found that the increase in SOD and CAT activities was positively correlated with cassava resistance to *T. cinnabarinus*. Then, the resistance of the reported transgenic cassava lines overproducing copper/zinc superoxide dismutase (*MeCu/ZnSOD*) and catalase (*MeCAT1*)^23^ to *T. cinnabarinus* was evaluated. The results of laboratory and field evaluation demonstrated that transgenic cassava lines were resistant to *T. cinnabarinus*, which directly and molecularly confirmed the role of SOD and CAT in cassava resistance to mite. These results will help in understanding the antioxidant defense responses in the cassava–mite interaction and molecular breeding of mite-resistant cassava to effectively control cassava pests.

## Results

### Change trends of SOD and CAT activities in cassava plants damaged by *T. cinnabarinus*

**Figure 1:**
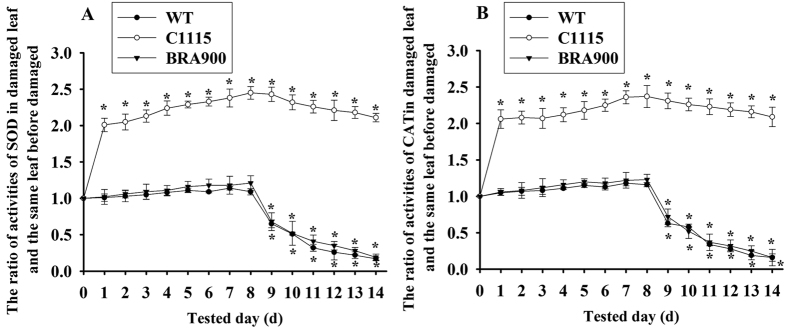
Change trend of SOD (**A**) and CAT (**B**) activities in different cassava cultivars damaged by *T. cinnabarinus*. The changes in SOD or CAT activities were presented as the ratio of the activities in 1 d to 14 d–damaged leaves and the same leaf before being damaged by *T. cinnabarinus* (0 d). Asterisk indicated activities of SOD and CAT in damaged leaves were significantly different from those in undamaged leaves (Student’s *t*-test, P < 0.05).

The change trend of SOD and CAT activities in cassava plants can reflect the oxidative burst kinetics damaged by *T. cinnabarinus*. As shown in Fig. 1, activities of SOD and CAT significantly increased in 1 d-damaged leaves and slowly increased to the maximum level which were 2.45 fold and 2.37 fold to those in undamaged leaves of C1115 respectively, and then decrease slowly but remained at a relatively high level in 9 d-damaged leaves to 14 d-damaged leaves (Student’s *t*-test, P < 0.05). However, activities of SOD and CAT in WT and BRA900 did not significantly change in 1 d-damaged leaves to 8 d-damaged leaves but significantly decreased in 9 d-damaged leaves to 14 d-damaged leaves compared with those in undamaged leaves. The results above indicated that 8 d was the optimal time for detecting the changes of SOD and CAT activities in different cassava cultivars damaged by *T. cinnabarinus.*

### Changes in SOD and CAT activities in different cassava cultivars damaged by *T. cinnabarinus*

**Figure 2:**
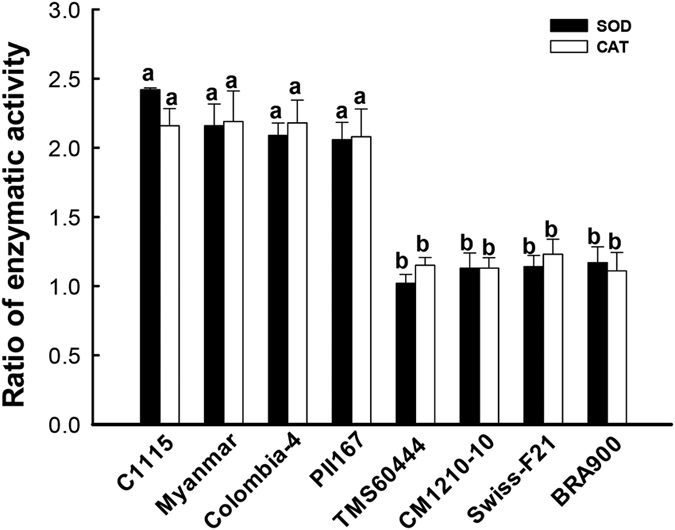
Changes in SOD and CAT activities in different cassava cultivars damaged by *T. cinnabarinus*. The changes in SOD or CAT activities were presented as the ratio of the activities in 8 d–damaged leaf and the same leaf before being damaged by *T. cinnabarinus*. Different letters above the standard error bars indicate significant differences in the activities of SOD or CAT in different cassava cultivars, which were based on the one-way ANOVA followed by Tukey’s HSD multiple comparison test (*P* < 0.05).

To understand the relationship between activities of SOD and CAT and cassava resistance to *T. cinnabarinus*, the changes in SOD and CAT activities were analyzed in different cassava cultivars damaged by *T. cinnabarinus*. It was found that the increase in SOD and CAT activities in mite-resistant cultivars was significantly different from the increase in mite-susceptible cultivars (Fig. 2). Compared with the activities in the same leaves before being damaged by *T. cinnabarinus*, the activities of SOD and CAT in 8 d–damaged leaves of mite-resistant cassava cultivars (C1115, Myanmar, Colombia-4, and PII167) significantly increased to approximately twofold, while the activities in 8 d–damaged leaves of mite-susceptible cassava cultivars (CM1210-10, SWISS-F21, BRA900, and WT) did not significantly change (the significance analysis was based on the one-way ANOVA followed by Tukey’s HSD multiple comparison test (*P* < 0.05)) (Fig. 2). Besides, the correlation coefficents of the increase in SOD and CAT activities and cassava resistance to *T. cinnabarinus* were 0.9631 and 0.9439 (*P* < 0.05), respectively. The aforementioned results indicated that cassava resistance to *T. cinnabarinus* was positively related to the increase in SOD and CAT activities.

### Molecular detection of transgenic cassava stability

**Figure 3:**
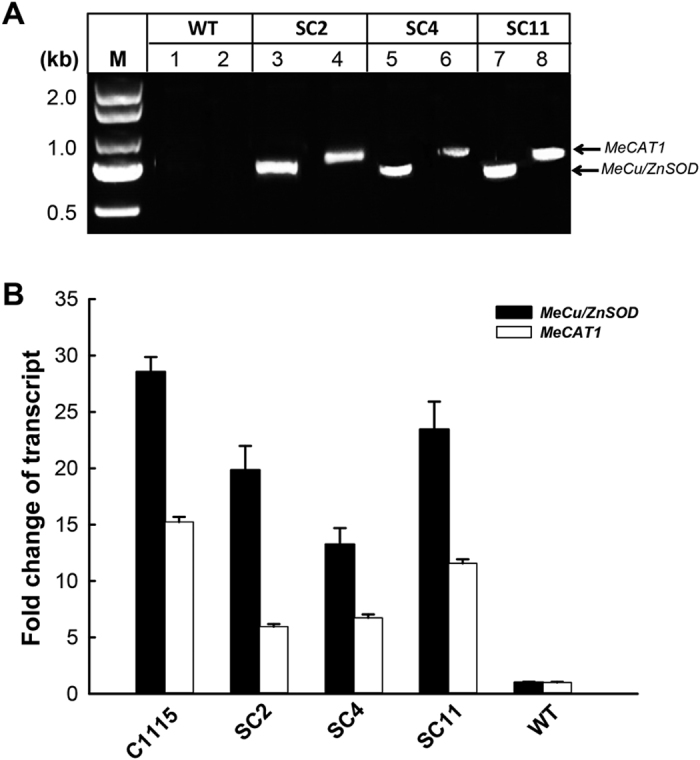
Molecular detection of transgenic cassava stability. (**A**) PCR products of *MeCu/ZnSOD* and *MeCAT1* as presented on 1% agarose gel. Lane M: DNA standard ladder (DL-2000); lanes 1, 3, 5, and 7: PCR products were amplified using genomic DNA of wild type (WT), SC2, SC4, and SC11 as templates, respectively, and primers were specific for *MeCu/ZnSOD*; lanes 2, 4, 6, and 8: PCR products were amplified using genomic DNA of WT, SC2, SC4, and SC11 as templates, respectively, and primers were specific for *MeCAT1.* (**B**) qRT-PCR analysis of transcript levels of *MeCu/ZnSOD* and *MeCAT1* in both transgenic cassava lines and WT. The relative transcript levels of *MeCu/ZnSOD* and *MeCAT1* in the WT were set as 1.0, and the transcript levels were presented as the ratios of *MeCu/ZnSOD* and *MeCAT1* in transgenic cassava lines, WT and C1115.

The same transgenic cassava lines^23^ were used to molecularly confirm their stability. PCR and qPCR were applied, and all the transgenic cassava lines were found to present PCR products of transgenes *MeCu/ZnSOD* (759 bp) and *MeCAT1* (823 bp), while the WT did not show any bands (Fig. 3A). In addition, compared with the levels in WT, the transcript levels of *MeCu/ZnSOD* and *MeCAT1* in transgenic cassava lines increased to 23- to 12-fold and 12- to 6-fold, and in C1115 increased to 28- and 15- fold, respectively (Fig. 3B). These results indicated the overexpression of *MeCu/ZnSOD* and *MeCAT1* in transgenic cassava lines.

### The tolerance of transgenic plants to oxidative stresses caused by *T. cinnabarinus*

**Figure 4:**
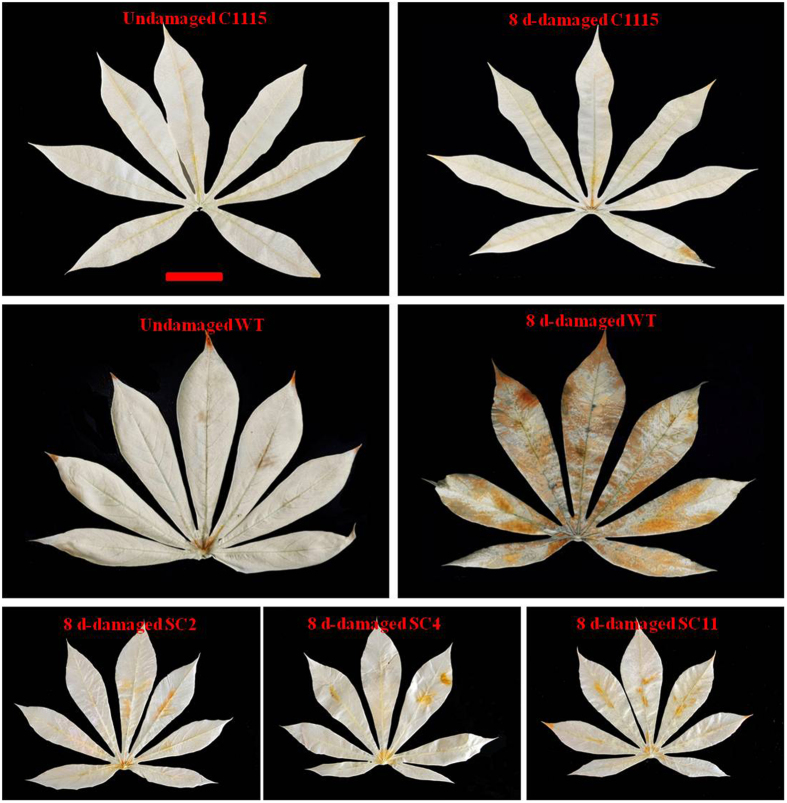
Enhanced tolerance to H_2_O_2_-mediated oxidative stress in different cassava plant leaves as detected by DAB staining. Bars = 5 cm.

3,3′-diaminobenzidine (DAB) staining was applied to confirm the enhanced ROS-scavenging capacity of the transgenic plants. Before inoculated with *T. cinnabarinus*, leaves of wild-type. C1115 and transgenic plants showed only a basal level of ROS (i.e., H_2_O_2_) production, but 8 d-damaged leaves of C1115 and transgenic plants showed much less H_2_O_2_ accumulation as evidenced by slight DAB staining, while 8 d-damaged wild-type leaves accumulated H_2_O_2_ significantly (Fig. 4). These results stated that the transgenic plants enhanced the tolerance to oxidative stresses caused by *T. cinnabarinus*.

### Laboratory evaluation of transgenic cassava resistance to *T. cinnabarinus*

#### Influence of transgenic cassava lines on the development and reproduction of T. cinnabarinus

**Figure 5:**
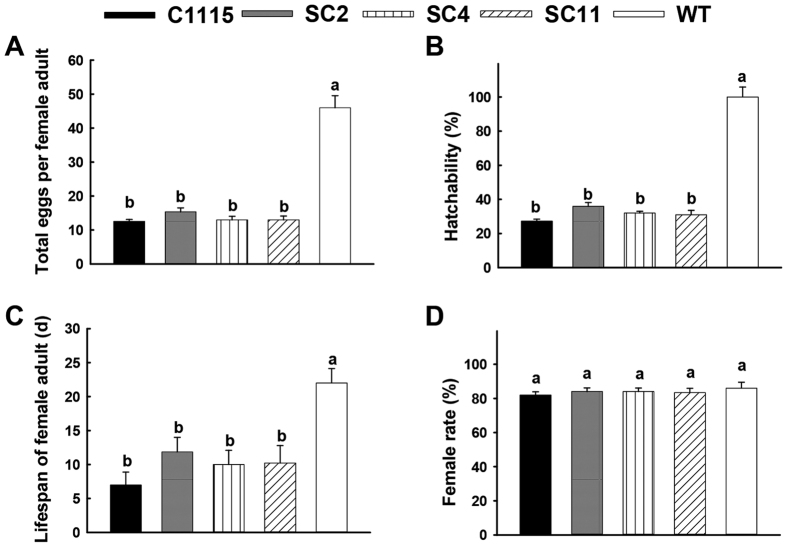
Influence of transgenic cassava lines on the development and reproduction of *T. cinnabarinus*. (**A**) Reproduction (total eggs per female adult). (**B**) Hatchability. (**C**) Lifespan of female adult. (**D**) Sex ratio (female rate). Different letters above the standard error bars indicate significant differences based on the one-way ANOVA followed by Tukey’s HSD multiple comparison test (*P* < 0.05).

**Figure 6:**
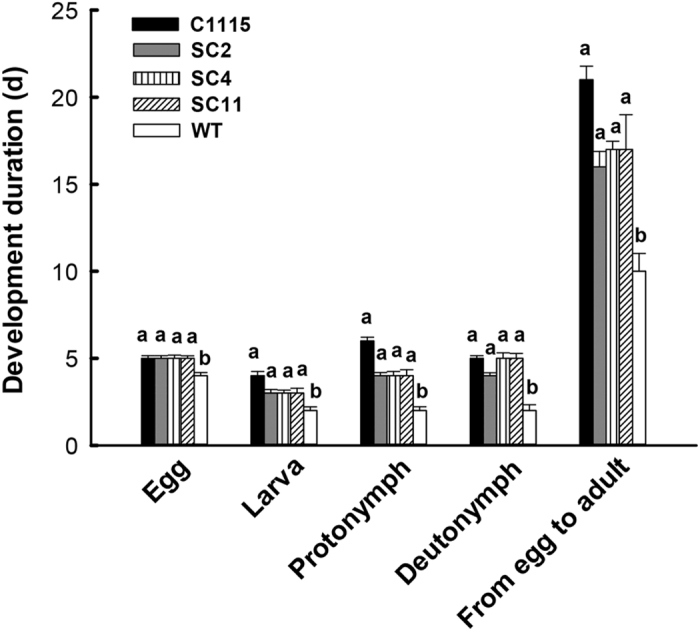
Duration of development period in *T. cinnabarinus* feeding on different cassava plants. Different letters above the standard error bars indicate significant differences among each life stage based on the one-way ANOVA followed by Tukey’s HSD multiple comparison test (*P* < 0.05).

The influence on the development and reproduction of *T. cinnabarinus* was important to evaluate the resistance of transgenic cassava lines to *T. cinnabarinus*. When *T. cinnabarinus* were fed on transgenic cassava lines SC2, SC4, and SC11, the total number of eggs per female was 15, 13, and 13, respectively, which were significantly less than the number of eggs in the mite-susceptible WT (46 eggs) and not significantly different from the number of eggs in the mite-resistant cassava control C1115 (12 eggs) (Fig. 5A). The egg hatchability was 36%, 32%, and 31%, respectively, which was significantly lower than the hatchability in WT (100%) and not significantly different from the hatchability in C1115 (25%) (Fig. 5B). The lifespans of female adults were 12, 10, and 10 d, respectively, which were significantly shorter than the lifespans in WT (22 d) and not significantly different from the lifespans in C1115 (7 d) (Fig. 5C). However, the sex ratios (female rate %) were 84%, 84%, and 83%, respectively, which were not significantly different from the sex ratios in WT (82%) and C1115 (86%) (Fig. 5D). Furthermore, the durations of development period of F_0_ *T. cinnabarinus* feeding on transgenic cassava lines were 5 (egg), 3 (larva), 4 (protonymph), 4–5 (deutonymph), and 16–17 d (from egg to adult), respectively, which were significantly longer than the durations in WT (4 d, egg; 2 d, larva; 2 d, protonymph; 2 d, deutonymph; 10 d, from egg to adult) and not significantly different from the durations in C1115 (6 d, egg; 4 d, larva; 6 d, protonymph; 5 d, deutonymph; 21 d, from egg to adult) (Fig. 6). These results suggested that the transgenic cassava lines could significantly inhibit the reproduction and development of *T. cinnabarinus*, which confirmed that the increase in SOD and CAT activities was positively related to cassava resistance to *T. cinnabarinus*.

#### Survival of T. cinnabarinus feeding on transgenic cassava lines

To understand transgenic cassava resistance to *T. cinnabarinus* in the laboratory, the survival of *T. cinnabarinus* feeding on transgenic cassava lines was tested. Compared with the mite-susceptible cassava control WT (88.9%), the survival of *T. cinnabarinus* F_0_ progeny was much lower in transgenic cassava lines SC2 (23.7%), SC4 (18.4%), and SC11 (26.9%) and in the mite-resistant cassava control C1115 (5.6%) (Table 1). Also, according to Technical regulations for the identification of cassava-germplasm resistance to pests^25^, transgenic cassava lines SC2, SC4, and SC11 were identified as “resistant”. These results together with the results shown in Figs 3 and 4 molecularly confirmed for the first time that the increase in SOD and CAT activities was positively related to cassava resistance to *T. cinnabarinus*.

#### Changes in SOD and CAT activities in T. cinnabarinus feeding on transgenic cassava lines

**Figure 7:**
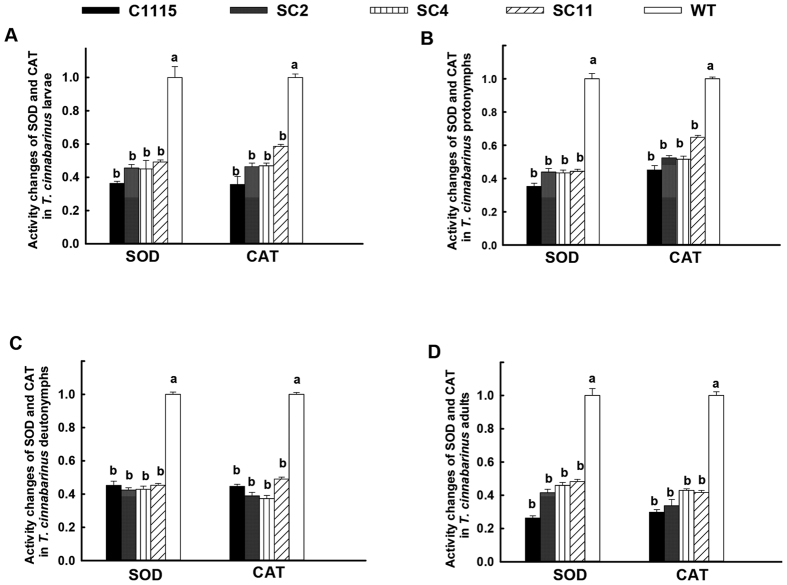
Changes in SOD and CAT activities in *T. cinnabarinus* feeding on transgenic cassava lines. (**A**) larvae, (**B**) protonymphs, (**C**) deutonymphs, and (**D**) adults. The activities of SOD or CAT in the WT were set as 1.0, and the changes in SOD or CAT activities were presented as the ratio of the activities in different developmental stages of *T. cinnabarinus* feeding on transgenic cassava lines and C1115 relative to those in *T. cinnabarinus* feeding on WT. Different letters above the standard error bars indicate significant differences based on the one-way ANOVA followed by Tukey’s HSD multiple comparison test (*P* < 0.05).

The changes in SOD and CAT activities in *T. cinnabarinus* feeding on transgenic cassava lines helped understand the inhibition of the development and reproduction of *T. cinnabarinus* by the transgenic cassava lines and the interaction between cassava and *T. cinnabarinus*. When feeding on transgenic cassava lines SC2, SC4, and SC11, the activities of SOD and CAT in larvae, protonymphs, deutonymphs, and adults of *T. cinnabarinus* were significantly lower than those in *T. cinnabarinus* feeding on WT (*P* < 0.05). The ratios of SOD activities in different developmental stages of *T. cinnabarinus* feeding on transgenic cassava lines relative to WT were listed as follows: 0.456, 0.450, and 0.492 for larvae (Fig. 7A); 0.439, 0.434, and 0.444 for protonymphs (Fig. 7B); 0.424, 0.429, and 0.453 for deutonymphs (Fig. 7C); and 0.415, 0.459, and 0.482 for adults (Fig. 7D), respectively. The ratios of CAT activities in different developmental stages of *T. cinnabarinus* feeding on transgenic cassava lines relative to WT were listed as follows: 0.464, 0.469, and 0.586 for larvae (Fig. 7A); 0.524, 0.516, and 0.648 for protonymphs (Fig. 7B); 0.389, 0.373, and 0.490 for deutonymphs (Fig. 7C); and 0.337, 0.429, and 0.417 for adults (Fig. 7D), respectively. Besides, the activities of SOD and CAT in different developmental stages of *T. cinnabarinus* feeding on transgenic cassava lines were not significantly different from those in *T. cinnabarinus* feeding on C1115. Moreover, a significant positive correlation was found between the resistance of transgenic cassava lines and the decrease in SOD (correlation coefficents for larvae, protonymphs, deutonymphs, and adults were 0.9454, 0.9356, 0.9436, and 0.9342, respectively) and CAT activities (correlation coefficents for larvae, protonymphs, deutonymphs, and adults were 0.9423, 0.9346, 0.9462, and 0.9435, respectively) in *T. cinnabarinus* feeding on transgenic cassava lines (*P* < 0.05). These data demonstrated that the transgenic cassava lines could significantly inhibit the activities of SOD and CAT in *T. cinnabarinus,* which also confirmed that the increase in SOD and CAT activities was positively related to cassava resistance to *T. cinnabarinus*.

#### Changes in SOD and CAT activities in transgenic cassava lines damaged by T. cinnabarinus

**Figure 8:**
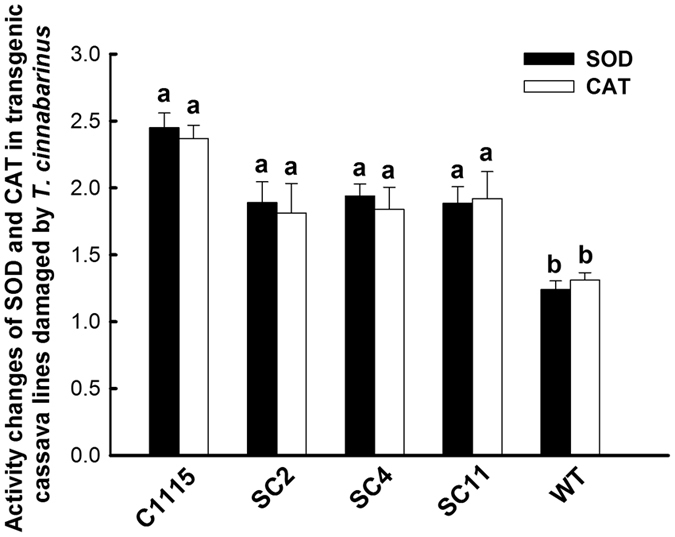
Changes in SOD and CAT activities in transgenic cassava lines damaged by *T. cinnabarinus*. The changes in SOD or CAT activities were presented as the ratio of the activities in 8 d–damaged leaf and the same leaf before being damaged by *T. cinnabarinus*. Different letters above the standard error bars indicate significant differences of activities of SOD or CAT among transgenic cassava lines, WT and C1115, which were based on the one-way ANOVA followed by Tukey’s HSD multiple comparison test (*P* < 0.05).

**Figure 9:**
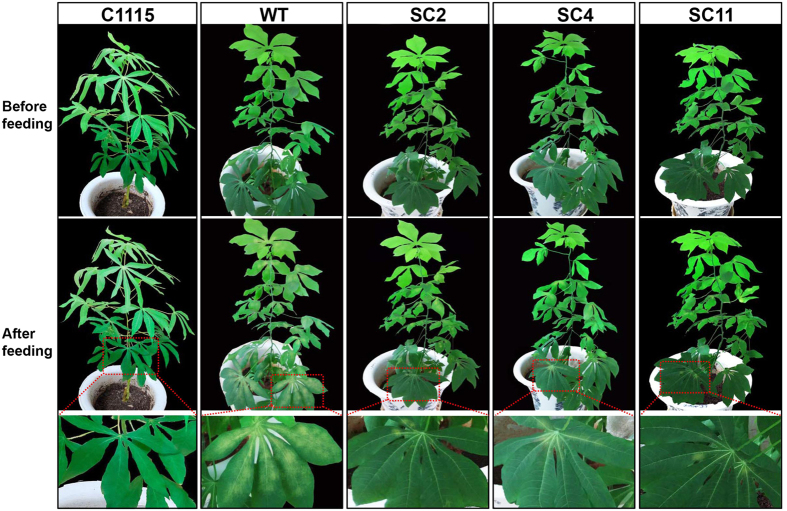
The symptoms in 3-month-old transgenic cassava lines damaged by *T. cinnabarinus* after 8 days in the laboratory. The bottom panels showed individual leaves. WT and C1115 were used as the mite-susceptible control and the mite-resistant control, respectively.

The changes in SOD and CAT activities in the damaged leaves were very important to evaluate the resistance of transgenic cassava lines to *T. cinnabarinus* and the interaction between cassava and *T. cinnabarinus*. The results showed that the activities of SOD and CAT in 8 d–damaged leaves of transgenic cassava lines SC2, SC4, and SC11 significantly increased (the significance analysis was based on the one-way ANOVA followed by Tukey’s HSD multiple comparison test (*P* < 0.05). The fold increases were listed as follows: SC2 (1.89- and 1.81-fold), SC4 (1.94- and 1.84-fold), and SC11 (1.86- and 1.92-fold). The increase in SOD and CAT activities in transgenic cassava lines was similar as the results in C1115 (2.45- and 2.37-fold). However, the activities of SOD and CAT in 8 d–damaged leaves of WT did not significantly change (Fig. 8). The correlation coefficents of the increase in SOD and CAT activities and cassava resistance to *T. cinnabarinus* were 0.9361 and 0.9525, respectively (*P* < 0.05). Furthermore, the significant increases in SOD and CAT activities in 8 d–damaged leaves of transgenic cassava lines were similar to the results in Fig. 2. In addition, after being damaged by *T. cinnabarinus* for 8 days, only few symptoms were found on the plants of the transgenic cassava lines SC2, SC4, and SC11, which was similar as C1115, while WT plants were seriously damaged (Fig. 9). These results further confirmed that the increased SOD and CAT activities were positively related to cassava resistance to *T. cinnabarinus*.

### Field evaluation of transgenic cassava resistance to *T. cinnabarinus*

**Figure 10:**
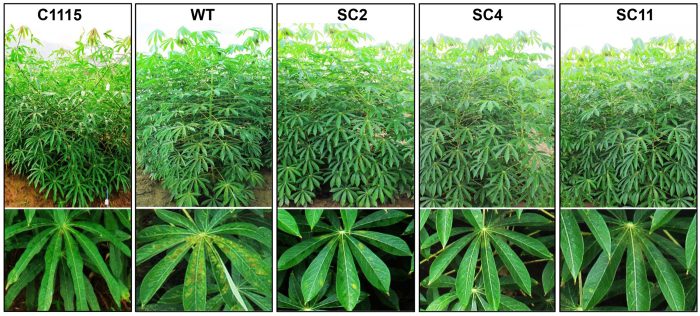
The damaged situtation in different cassava plants in the field. The bottom panels showed individual leaves. WT and C1115 were used as the mite-susceptible control and the mite-resistant control, respectively.

Field tests were conducted to evaluate cassava resistance to *T. cinnabarinus.* Compared with the mite-resistant cassava control C1115 (0.6%) and the mite-susceptible WT (96.6%), the mite damage indexes for transgenic cassava cultivars SC2, SC4, and SC11 were 19.8%, 18.2%, and 20.9%, respectively (Table 2), which was identified as “resistance” to *T. cinnabarinus.* Furthermore, only few symptoms were observed on the plants of the transgenic cassava lines SC2, SC4, and SC11, which was similar as C1115, while WT plants were seriously damaged by *T. cinnabarinus* (Fig. 10); the symptoms were similar to the symptoms shown in Fig. 9. The results of field evaluation and laboratory evaluation directly and convincingly validated that the transgenic cassava lines overproducing *MeCu/ZnSOD* and *MeCAT1* were resistant to *T. cinnabarinus*, and the increase in SOD and CAT activities contributed to cassava resistance to *T. cinnabarinus*.

## Discussion

Protective enzymes are involved in plant defense responses to pests^11^. The ROS generated by pests can be scavenged by protective enzymes, which can result in the effective reduction of oxidative burst^10^. Among these protective enzymes, SOD acts as the first line of defense against ROS^26^, and its enzymatic action results in the formation of H_2_O_2_. CAT then detoxifies the decomposition of H_2_O_2_ to water and oxygen^27^. Therefore, the enhanced activities of SOD and CAT were considered to be closely related to plant resistance to pests. The studies conducted in cucumber^11^, pea^12^, rice^28^, and citrus^13^ suggested that the increase in SOD and CAT activities accounted for plant resistance to pests. Similarly, we found the activities of SOD and CAT gradually increased to the maximum level in 8 d-damaged leaves of mite-resistant cassava cultivars (Fig. 1). Moreover, the transgenic plants overexpressing SOD and CAT enhanced the tolerance to oxidative stresses caused by *T. cinnabarinus* as evidenced by DAB staining (Fig. 4). These results illustrated the important role of SOD and CAT in plant resistance to pests, which needs to be molecularly confirmed.

The downregulation of endogenous SOD and CAT would significantly inhibit the development and reproduction of pests. Deletion of SOD genes in yeast^29^, flies^29^,^30^, and mice^31^,^32^ resulted in a decreased lifespan. In this study, the lifespan of female adult *T. cinnabarinus* significantly decreased (Fig. 5C), which was possibly due to the suppression of SOD in *T. cinnabarinus* feeding on the transgenic cassava lines. Moreover, a trade-off was observed between self-maintenance and reproduction in animals. The effects of such a trade-off on the oxidative status depend on whether priority is given to self-maintenance or reproduction^33^. Accordingly, the development and reproduction of *T. cinnabarinus* feeding on the transgenic cassava lines were significantly inhibited in this study (Figs 5 and 6). It was speculated that the downregulation of endogenous SOD and CAT would be expected in inhibiting the reproduction of *T. cinnabarinus* for self-maintenance, which would cause the decrease in the total number of eggs per female adult and hatchability, and the prolongation of the development period.

The activity analysis of SOD and CAT helped in understanding the interaction between plant and pests. On the one hand, pests can seriously disrupt normal metabolism through oxidative damage to lipids, protein, nucleic acids, and cells of plant leaves, which can cause plant cell death and chlorosis^10^. On the other hand, the SOD activity increases initially (as the first line of defense) to transform ROS into H_2_O_2_; the increase in substrate H_2_O_2_ subsequently elevates the activity of CAT, which detoxifies H_2_O_2_ to water and oxygen^27^. In this study, the activities of SOD and CAT in *T. cinnabarinus* feeding on transgenic cassava lines SC2, SC4, and SC11 were found to be significantly lower than those in *T. cinnabarinus* feeding on the control WT (Fig. 7). Moreover, the activities of SOD and CAT in transgenic cassava lines SC2, SC4, and SC11 damaged by *T. cinnabarinus* significantly increased, while they did not significantly change in the WT (Fig. 8). These results demonstrated that the overproduction of SOD and CAT in transgenic cassava lines could coordinately activate the ROS scavenging machinery to reduce the oxidative damage and significantly enhance the resistance of transgenic cassava lines to *T. cinnabarinus*, while the decrease in SOD and CAT activities significantly inhibited the reproduction and development of *T. cinnabarinus*, which resulted in a few symptoms on the plants of the transgenic cassava lines SC2, SC4, and SC11 as C1115 but caused serious damage on the WT (Figs 9 and 10).

In summary, the results of laboratory and field evaluation in this study directly and convincingly confirmed the function of *MeCu/ZnSOD* and *MeCAT1* overproduction in cassava resistance to *T. cinnabarinus*, in addition to their role in abiotic stress improvement (Xu *et al*., 2013, 2014). As Taylor *et al*.^34^ speculated, future transgenic programs will involve the transfer of beneficial traits between resistant and susceptible cassava varieties. This study provided an example to construct transgenic cassava lines by using inherent protective enzyme genes *MeCu/ZnSOD* and *MeCAT1*, which would be much more helpful for understanding the mechanism of plant resistance to pests and molecular breeding of mite-resistant cassava for effective pest control.

## Methods

### *T. cinnabarinus* rearing

Healthy *T. cinnabarinus* adults were maintained by the Environment and Plant Protection Institute, Chinese Academy of Tropical Agricultural Sciences (CATAS), and reared on the back of healthy cassava leaves under the following conditions: temperature 28 ± 2 °C, 75 ± 5% relative humidity, and 14 L:10 D photoperiod. Healthy cassava leaves of similar growth status were collected from 10–16 leaves below the apical bud of 6-month-old plants in the field. A water-saturated blotting paper strip was wrapped around the leaf margin to prevent the escape of mites and keep the leaf fresh. The leaves were replaced every 2 days.

### Cultivation of cassava cultivars and transgenic cassava lines

Resistant cassava cultivars to *T. cinnabarinus* C1115, Myanmar, Colombia-4, and PII167 and susceptible cultivars to *T. cinnabarinus* CM1210-10, SWISS-F21 and BRA900 were supplied by National Cassava Germplasm Nursery of China, CATAS. The susceptible cultivar to *T. cinnabarinus* TMS60444, served as wild type (WT), and the reported transgenic cassava lines SC2, SC4, and SC11^23^ were supplied by the Institute of Plant Physiology and Ecology, Chinese Academy of Sciences. All cassava cultivars and transgenic cassava lines were planted in pots (33 cm in diameter × 25 cm in height) containing 5 kg of well-mixed soil (soil: peat: perlite = 1:1:1) and grown in the greenhouse (14 h/10 h of light/dark, 28 °C ± 2 °C of day/night). After 3 months, some cassava plants were selected to test the transcription and enzyme activities of SOD and CAT. In addition, other cassava plants were transplanted into the field, and then 6-month-old leaves were selected to rear *T. cinnabarinus* and examine the effects on their survival, reproduction, development, and activities of SOD and CAT in the laboratory. Besides, the field investigation of cassava resistance capacity to *T. cinnabarinus* was also based on 6-month-old cassava plants.

### Activity tests of SOD and CAT

To test the activities of SOD and CAT in cassava plants, three leaves from below the apical bud of the 3-month-old laboratory cassava plants were inoculated with 50 female adults of *T. cinnabarinus*. To evaluate the change trend of activities of SOD and CAT in cassava cultivars WT, C1115 and BRA900, adults were removed and 100 mg of damaged leaves were used for SOD and CAT extraction (100 mg of undamaged leaves were used as controls). Continuous sampling was conducted from 1 d to 14 d in order to find out the optimal timepoint which was applied to collect the samples for subsequent examination of SOD and CAT activities in cassava plants.

To test the activities of SOD and CAT in *T. cinnabarinus* feeding on transgenic cassava lines, each leaf collected from healthy 6-month-old leaves in the field was inoculated with 200 female adults. After 24 h, the adults were removed and the eggs were kept and reared; 600 larvae, 600 protonymphs, 600 deutonymphs, and 200 female adults of F_0_ *T. cinnabarinus* were collected for SOD and CAT extraction. All the experiments were carried out in triplicate. The samples were transferred into a prechilled mortar and thoroughly triturated in extract solution before centrifuging at 10,000 rpm for 10 min at 4 °C. The supernatant was collected and mixed with precooling distilled water to 10 mL.

The SOD activity was determined by a modified method of pyrogallic acid autoxidation^35^. The autoxidation rate was determined by the following procedure: at 25 °C, 10 mL of 50 μL/L pyrogallic acid was mixed with 4.5 mL of 50 mmol/L, pH 8.3, K_2_HPO_4_–KH_2_PO_4_ buffer. The absorbence was measured at the wavelength of 325 nm every 30 s, and the autoxidation rate was required to be controlled at 0.070 OD/minute. The SOD activity was measured when the pyrogallic acid was added to the mixture containing 10 μL of the sample (V2) and 4.5 mL of K_2_HPO_4_–KH_2_PO_4_ buffer, and the absorbence (A) of the mixture at 325 nm was recorded every 30 s. The activity of SOD was calculated by the following formula:


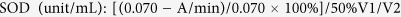


V1: volume of the final mixture, mL

V2: volume of the sample, mL

The catalase (CAT) activity was determined by the consumption of H_2_O_2_ according to the method described by Zhang *et al*.^36^. The reaction mixture (1.5 mL) contained 50 mM potassium phosphate buffer (pH 7.0), 15 mM H_2_O_2_, and 100 μL of the enzyme extract. The consumption of H_2_O_2_ was monitored spectrophotometrically at 240 nm and quantified by its molar extinction coefficient (36 mol/Lcm). The CAT activity was presented as CAT units per minute per milligram of protein.

### Molecular detection of transgenic cassava stability

To test the stability of transgenic cassava lines SC2, SC4, and SC11 overproducing SOD and CAT, genomic DNA were isolated from three-month-old leaves of transgenic cassava lines SC2, SC4 and SC11 and the control WT as described previously^37^ and analyzed for the gene characterization of *MeCu/ZnSOD* and *MeCAT1* by polymerase chain reaction (PCR). Besides, total RNA was isolated from 3-month-old leaves of transgenic cassava lines and the WT using TRIzol reagent (Invitrogen, CA, USA) and first treated with gDNA Eraser (Takara Biochemicals, Dalian, China) to remove genomic DNA contamination. Then, 1.0 μg of total RNA was used for cDNA synthesis with a First Strand cDNA Synthesis Kit (Fermentas Inc, MD, USA), and oligo (dT)_18_ was used as primer. All the experiments were carried out in triplicate.

Gene characterization of *MeCu/ZnSOD* and *MeCAT1* was performed by PCR using the reported primers (Table 3; one primer bound to the promoter region and another bound to the gene region)^23^ under the following conditions: 95 °C for 5 min, 30 cycles of 95 °C for 1 min, 57 °C for 1 min, and 72 °C for 1 min, and a final extension at 72 °C for 10 min. Finally, PCR products of *MeCu/ZnSOD* and *MeCAT1* were separated on a 1.0% agarose gel and visualized by Goldview staining.

The transcriptional characterization of *MeCu/ZnSOD* and *MeCAT1* was performed by quantitative real-time PCR (qRT-PCR). Cassava *β-actin* was used as a reference for normalization. The cDNA product from the reverse transcription was tenfold diluted, and 2 μL of each diluted sample was used for qPCR with a SYBR Green PCR kit (Takara Biochemicals, Dalian, China) and the reported primers (Table 3)^23^ and analyzed on an ABI StepOnePlus Real-Time PCR System (PerkinElmer Applied Biosystems, CA, USA). PCR amplification was conducted by a 1-min preincubation step at 95 °C followed by 40 cycles of 95 °C for 15 s, 60 °C for 15 s, and 72 °C for 20 s. The relative transcript levels of *MeCu/ZnSOD* and *MeCAT1* were calculated according to the 2^−△△Ct^ method^38^.

### DAB Staining

DAB staining was performed according to the method reported by Thordal *et al*.^39^. Undamaged and 8 d damaged leaves by *T. cinnabarinus* (three-month-old) were infiltrated with 10 mL of DAB solution (1 mg/mL DAB, pH 3.8) for 8 h. Leaves were immersed in 95% (w/v) boiling ethanol for 10 min to decolorize the chloroplast.

### Laboratory and field evaluation of transgenic cassava resistance to *T. cinnabarinus*

The laboratory evaluation of cassava resistance capacity to *T. cinnabarinus* depended not only on the survival, development, and reproduction of *T. cinnabarinus* feeding on transgenic cassava plants, but also on the changes in SOD and CAT activities in both cassava leaves and *T. cinnabarinus*.

To examine the influence of transgenic cassava lines SC2, SC4, and SC11 on survival, reproduction, and development of *T. cinnabarinus*, 10 pairs of adults per leaf were inoculated on the back of the leaf from 6-month-old plants in the field. Fifty leaves per transgenic cassava lines were selected for the survival test and 9 leaves per transgenic cassava lines for reproduction and development of *T. cinnabarinus.* After 24 h, the adults were removed and eggs were kept and reared. The egg hatchability; survival; duration of development period of egg, larva, protonymph, and deutonymph; and lifespan of female adults were recorded every 8 h, and the total eggs per female adults, egg hatchability, develoment duration, sex ratio, lifespan of female adults, and survival of F_0_ *T. cinnabarinus* were calculated. The resistance of transgenic cassava lines to *T. cinnabarinus* were evaluated according to Technical regulations for the identification of cassava-germplasm resistance to pests^25^ (Table 4). WT and C1115 were used as the mite-susceptible control and the mite-resistant control, respectively.

The activity analyses of SOD and CAT in transgenic cassava lines, WT, C1115 and *T. cinnabarinus* were performed according to the method mentioned in the “Activity tests of SOD and CAT” section.

Field evaluation was also based on Technical regulations for the identification of cassava-germplasm resistance to pests^25^. Based on the symptoms due to damage by *T. cinnabarinus*, the damage scale of cassava leaf was classified into five scales as follows:

0: No damage, healthy growth of the leaf

1: The mite-damaged area accounts for less than 25% of the whole leaf

2: The mite-damaged area accounts for 26–50% of the whole leaf

3: The mite-damaged area accounts for 51–75% of the whole leaf

4: The mite-damaged area accounts for more than 76% of the whole leaf.

The evaluation standard of cassava resistance to *T. cinnabarinus* in the field was classified into six levels (Table 5).

### Statistical analysis

Data were statistically analyzed using the SPSS software (version 16.0; IL, USA) and was expressed as the mean ± standard error of the mean. One-way analysis of variance (ANOVA) was applied to calculate statistical significance followed by Tukey’s honestly significant difference (HSD) multiple comparison test (*P* < 0.05). A *P* value less than 0.05 was considered statistically significant. Correlation analysis was also based on SPSS.

## Additional Information

**How to cite this article**: Lu, F. *et al*. Overproduction of superoxide dismutase and catalase confers cassava resistance to *Tetranychus cinnabarinus. Sci. Rep.* **7**, 40179; doi: 10.1038/srep40179 (2017).

**Publisher's note:** Springer Nature remains neutral with regard to jurisdictional claims in published maps and institutional affiliations.

## Figures and Tables

**Table 1:** Resistance identification of transgenic cassava lines to *T. cinnabarinus* in the laboratory.

Cassava genotype	F_0_ progeny survival rate (%)	Resistance level
SC2	23.7	R
SC4	18.4	R
SC11	26.9	R
C1115	5.6	HR
TMS60444 (WT)	88.9	HS

**Table 2:** Resistance identification of transgenic cassava lines to *T. cinnabarinus* in the field.

Cassava genotype	Mite damage index (%)	Resistance level
SC2	19.8	R
SC4	18.2	R
SC11	20.9	R
C1115	0.5	HR
TMS60444 (WT)	96.6	HS

**Table 3:** Primers used for PCR and qRT-PCR detection of transgenic cassava lines.

Application	Primer name	Sequence (5′—3′)	*T*_m_ (°C)	Product length (bp)
PCR	MeCu/ZnSOD-F	ATGGCCCTCCATTATTTACACT	57.5	759
	MeCu/ZnSOD-R	AACAACGACTGCCCTTCCTACAAT	62.0	
	MeCAT1-F	TGTGAAGATAGTGGAAAAGGAAGG	59.5	823
	MeCAT1-R	CTCAGGATGGTGTGATAAGAAGTC	58.0	
qRT-PCR	MeCu/ZnSOD(Q)-F	ATGTTCATGCCCTTGGAGAC	58.3	114
	MeCu/ZnSOD(Q)-R	GATCACCAGCATGACGAATG	58.4	
	MeCAT1(Q)-F	TGGGAAACAACTTCCCTGTC	59.4	172
	MeCAT1(Q)-R	ACATCATCGAAGAACCAGGC	58.5	
	Meactin(Q)-F	TGATGAGTCTGGTCCATCCA	59.8	108
	Meactin(Q)-R	CCTCCTACGACCCAATCTCA	60.2	

**Table 4:** Evaluation standard of cassava resistance to *T. cinnabarinus* in the laboratory.

Cassava resistance level	F_0_ progeny survival rate (%)
Immunity (IM)	0.0
Highly Resistant (HR)	0.1–10.0
Resistant (R)	10.1–30.0
Moderate Resistant (MR)	30.1–60.0
Susceptible (S)	60.1–80.0
High Susceptible (HS)	>80.0

**Table 5:** Evaluation standard of cassava resistance to *T. cinnabarinus* in the field.

Cassava resistance level	I (%)
Immunity (IM)	0.0
Highly Resistant (HR)	0.1–12.5
Resistant (R)	12.6–37.5
Moderate Resistant (MR)	37.6–62.5
Susceptible (S)	62.6–87.5
High Susceptible (HS)	>87.5
